# Profiling of differentially expressed genes in sheep T lymphocytes response to an artificial primary *Haemonchus contortus* infection

**DOI:** 10.1186/s13071-015-0844-z

**Published:** 2015-04-18

**Authors:** Yi Yang, Qian-Jin Zhou, Xue-Qiu Chen, Bao-Long Yan, Xiao-Lu Guo, Hong-Li Zhang, Ai-Fang Du

**Affiliations:** Institute of Preventive Veterinary Medicine, College of Animal Sciences, Zhejiang University, Hangzhou, 310058 China; Zhejiang Provincial Key Laboratory of Preventive Veterinary Medicine, Zhejiang University, Hangzhou, 310058 China; Faculty of Life Science and Biotechnology, Ningbo University, Ningbo, 315211 China; Wenzhou Medical University, Wenzhou, 325035 China; Zhejiang center for animal disease control and prevention, Hangzhou, 310000 China

**Keywords:** Sheep T lymphocytes, *Haemonchus contortus*, Microarray, Differentially expressed genes

## Abstract

**Background:**

*Haemonchus contortus* is a common bloodsucking nematode causing widespread economic loss in agriculture. Upon *H. contortus* infection, a series of host responses is elicited, especially those related to T lymphocyte immunity. Existing studies mainly focus on the general immune responses of sheep T lymphocyte to *H. contortus*, lacking investigations at the molecular level. The objective of this study was to obtain a systematic transcriptional profiling of the T lymphocytes in *H. contortus* primary-infected sheep.

**Methods:**

Nematode-free sheep were orally infected once with *H. contortus* L3s. T lymphocyte samples were collected from the peripheral blood of 0, 3, 30 and 60 days post infection (dpi) infected sheep. Microarrays were used to compare gene transcription levels between samples. Quantitative RT-PCR was employed to validate the microarray data. Gene Ontology and KEGG pathway analysis were utilized for the annotation of differentially expressed genes.

**Results:**

Our microarray data was consistent with qPCR results. From microarrays, 853, 242 and 42 differentially expressed genes were obtained in the 3d vs. 0d, 30d vs. 0d and 60d vs. 0d comparison groups, respectively. Gene Ontology and KEGG pathway analysis indicated that these genes were involved in metabolism, signaling, cell growth and immune system processes. Functional analysis of significant differentially expressed genes, such as SLC9A3R2, ABCB9, COMMD4, SUGT1, FCER1G, GSK3A, PAK4 and FCER2, revealed a crucial association with cellular homeostasis maintenance and immune response. Our data suggested that maintaining both effective immunological response and natural cellular activity are important for T lymphocytes in fighting against *H. contortus* infection.

**Conclusions:**

Our results provide a substantial list of candidate genes in sheep T lymphocytes response to *H. contortus* infection, and contribute novel insights into a general immune response upon infection.

**Electronic supplementary material:**

The online version of this article (doi:10.1186/s13071-015-0844-z) contains supplementary material, which is available to authorized users.

## Background

*Haemonchus contortus* is an important ruminant gastrointestinal nematode, causing diarrhea, anemia, emaciation, and death in serious infection [1]. Haemonchosis results in a significant global economic loss in the farming industry every year. Whilst chemical control is the main strategy [2], evolution of drug resistance and residues severely reduces the success of anthelmintic treatment programs [3,4]. New control strategies, such as immunological protective method, are urgently in need and are being developed rapidly. At the same time, approaches to better understand the underlying mechanisms of host immune response to *H. contortus* infection require rapid implementation.

In general, the developmental process of *H. contortus* in the host involves a few steps [5]. Infective third-stage larvae (L3s) become exsheathed and develop into L4s after oral infection by the host. After that L4s arrive at the abomasums ready to parasitize at 3–5 days post infection (dpi). Before reaching adulthood, they need to escape from the host’s immune system, avoiding host’s rejection. Subsequently the adults begin to lay eggs at approximately 18 dpi and the parasitic load peak by 25–30 dpi. Finally, *H. contortus* reaches a stable parasitic level upon achieving immune evasion in its host.

To expel the nematodes during such infection, the host relies mainly on T lymphocytes, especially T helper 2 (TH2) cells [6,7]. The TH2-type immune response induces the production of the various cytokines, such as IL-4, IL-5, IL-10, IL-13, IL-25 and IL-31 [8]; contributes to B cell differentiation leading to the expression of antibodies, such as IgE, IgG1, IgG4 and IgA [9,10]; and gathers eosinophils to target and eradicate the nematodes [11]. In addition, the TH2-type immune response can reduce T helper 1 (TH1)-type immune response-mediated pathological inflammation through cross suppression, a reaction that further challenges nematode survival [12-14]. During such immune responses, T lymphocytes also mobilize factors involved in cell homeostasis to maintain a stable environment for the cells, ensuring the immune system in optimum working conditions.

A number of studies have described the general aspects of sheep T lymphocyte immune responses to *H. contortus* [15,16], while Nicholas *et al.* examined gastrointestinal tract and lymph node tissues and gene expression associated with *H. contortus* resistance in sheep [17]. Thus, we speculated that obvious changes in gene expression profiles of T lymphocytes upon *H. contortus* infection must exist. We hypothesized that these differentially expressed genes are likely to participate in T lymphocyte immune responses and/or cell homeostasis maintenance. This study therefore aims to obtain the profiles of differentially expressed genes in the sheep T lymphocytes in response to *H. contortus* infection to increase our current understanding of sheep immune response to *H. contortus*.

## Methods

### Animals and parasites

Five six-month-old female Hu sheep were raised in nematode-free conditions and handled humanely in strict accordance with the Zhejiang University Guidance for care and use of experimental animals (Zhejiang University, Code ZJU201308-1-10-072). Four sheep were orally infected with 17,000 *H. contortus* L3s, and one was kept as an uninfected control. Infective larvae of *H. contortus* ZJ strain were cultured from eggs using standard methods. The eggs were collected from naturally singly-infected sheep.

### Sampling

Peripheral blood samples of each sheep were collected in sodium citrate coated tubes at 0, 3, 30 and 60 dpi. T lymphocytes were then separated using lymphocyte separation medium (Huadong Medicine), purified through nylon wool fiber [18-20] and resuspended in TRIzol reagent (Invitrogen). Samples were preserved in liquid nitrogen until RNA preparation.

### RNA preparation

Total RNA of T lymphocyte samples were obtained using TRIzol reagent (Invitrogen) following the manufacturer’s instructions and further purified using RNeasy Mini kit (Qiagen). RNA quantity and quality were determined using an Infinigen SSP-3300 ultramicro-spectrophotometer and an Agilent 2100 Bioanalyzer respectively. T lymphocytes RNA samples from three sheep were chosen for microarray experiments based on their good quantity and quality measurement. cRNA of these twelve RNA samples was then synthesized, labeled with Cy3 and used for hybridization.

### Microarray experiments

Sheep draft genome sequences and supplementary sequences from other species such as *Bos taurus* [21] were acquired from GenBank to prepare a microarray [17]. An Agilent sheep microarray containing 19,075 UniGene probes, 25,865 expressed sequence tags (ESTs, blast against UniGene, e value > 0.001) and 1,417 control probes were used in this experiment (Biostar Genechip INC., Shanghai). The GEO accession number of the microarray platform is GPL16283 [22], while that of the microarray series is GSE42302 [23]. The various Cy3 labeled cRNA samples were hybridized to the respective microarrays [24]. Fluorescence signals were scanned by Agilent scanistor with scan resolution 5 μm, PMT 100%.

### Data statistics and analysis

Feature Extraction software was used to read the raw data. The raw data files were processed using the Agi4x44Preprocess package based on linear models for microarray data (limma) package developed within the Bioconductor project in the R statistical programming environment. Background correction and normalization, probes filtering and replicated probes merger were included in data pre-processing to generate microarray results for gene expression profiling. In order to eliminate errors caused by one-sample analysis, the microarray results of three infected sheep of the same stage were combined to proceed to multi-sample analysis using limma package (P < 0.05), which were designated in groups as the 0d group, 3d group, 30d group and 60d group. Genes with absolute log2 fold change greater than 1 (P < 0.05) in pair-wise comparison between groups were scored as differentially expressed genes in response to *H. contortus* infection.

Functional categories of differentially expressed genes were classified with the bioinformatics analysis resource Database for Annotation, Visualization and Integrated Discovery (DAVID, http://david.abcc.ncifcrf.gov/). Gene Ontology (GO), KEGG pathway and PANTHER pathway were used in DAVID for gene function clustering [25].

### Quantitative RT-PCR (qPCR) validation

Five immune-related genes were randomly selected for reverse transcription PCR [26] to validate our microarray data and beta-actin was used as an internal control. Primers used for amplification are shown in Table 1. The cDNA was synthesized from the same RNA sample used in the initial microarray experiment. Quantitative RT-PCR assay was performed using SYBR Green system (Toyobo). Relative expression was calculated by the △Ct method, normalizing Ct value of each gene to beta-actin, followed by comparing the 3d, 30d, 60d versus 0d results of each sheep respectively.

**Table 1:** **Primers of ovine beta-actin and five immune-relevant genes selected for quantitative RT-PCR validation**

**Acronym**	**Gene name**	**Primer sequence (5′ – 3′)**	**GenBank number**	**Product size (bp)**
Beta-actin	Beta-actin	GGCAGGTCATCACCATCGGCAAT GCGTAGAGGTCTTTGCGGATGT	U39357.1	151
IL-8	Interleukin 8	AAAAGTGGGTGCAGAAGG CTCAAGGCACTGAAGTAGAT	NM_001009401.1	163
FCER1G	Fc fragment of IgE, high affinity I, receptor for; gamma polypeptide	GACCCAGGAGACTTATGAGACC GCGTATGTGATGCCAACC	NM_174537.2	123
IGLL1	immunoglobulin lambda-like polypeptide 1	CTCCAAACAGAGCAACAGC TGAGGGCTTCACTGTCTTC	NM_001083800.1	136
CXCL2	chemokine (C-X-C motif) ligand 2	AGGACTTGATGTGCTGGACT CAGGACTGGGTTATGTTTG	NM_174299.2	139
OVAR	MHC class I antigen	TGTTGCGGAGGCAGAAAGG ATGTGCCTTTGGAGGGTCTGC	NM_001130934.1	112

## Results

### Analysis of global gene expression

Six datasets of multi-sample analysis in pair-wise comparison between groups were obtained. They were 3d vs. 0d, 30d vs. 0d, 60d vs. 0d, 30d vs. 3d, 60d vs. 3d and 60d vs. 30d. Table 2 presents an overall view of the differentially expressed genes in the six comparisons. A total of 853 (99 up-regulated), 242 (234 up-regulated), 42 (15 up-regulated), 1058 (977 up-regulated), 805 (689 up-regulated) and 102 (2 up-regulated) differentially expressed genes were acquired in the 3d vs. 0d, 30d vs. 0d, 60d vs. 0d, 30d vs. 3d, 60d vs. 3d and 60d vs. 30d comparisons, respectively. Details of these genes are presented in Additional file 1. Thirty-five genes were common in the 3d vs. 0d, 30d vs. 0d and 60d vs. 0d comparisons (Table 3), with the functions of half of them remained unknown.

**Table 2:** **General situation of differentially expressed genes in six comparisons**

**Database**		**3d vs. 0d**		**30d vs. 0d**		**60d vs. 0d**		**30d vs. 3d**		**60d vs. 3d**		**60d vs. 30d**	
Diff genes	Up-regulated	853	99	242	234	42	15	1058	977	805	689	102	2
	Down-regulated		754		8		27		81		116		100
GO annotation		327		71		14		389		320		37	
KEGG Pathway annotation		139		26		9		159		139		11	
PANTHER Pathway annotation		41		12		2		60		46		7	

Summary of the amount and annotations of differentially expressed genes (diff genes) in T lymphocytes of sheep infected with *H. contortus* in the six comparisons.

**Table 3:** **Common thirty-five genes differentially expressed in the 3d vs. 0d, 30d vs. 0d and 60d vs. 0d comparisons**

**Gene symbol**	**Gene name**	**GenBank number**	**Log fold change**		
			**3d vs. 0d**	**3d vs. 0d**	**3d vs. 0d**
LOC100028054	similar to A kinase (PRKA) anchor protein (yotiao) 9	DY498437.1	−1.8385	2.803	1.744
-	-	CF116320.1	−1.4735	1.6095	1.1825
-	-	EE858261.1	−2.338	1.746	1.2545
-	-	EE823315.1	−1.4945	2.236	2.0075
-	-	EE782465.1	−2.3485	−1.6385	−1.97
-	-	GO684492.1	−1.6265	−1.444	−1.2705
SLC9A3R2	solute carrier family 9 (sodium/hydrogen exchanger), member 3 regulator 2	DY520662.1	−1.1435	2.878	2.3875
SCN8A	sodium channel, voltage-gated, type VIII, alpha	EE862637.1	−2.443	1.2955	1.0355
-	-	GO758506.1	−1.906	−1.005	−1.3805
-	-	DY500374.1	−1.4935	−1.389	−1.316
LOC100057304	similar to Chromosome 1 open reading frame 2	DY491988.1	−2.869	1.2115	1.037
RXRB	retinoid X receptor, beta	EE807201.1	1.5525	2.3845	1.0745
-	-	EE867875.1	−1.7765	−1.206	−1.442
NUDT14	nudix (nucleoside diphosphate linked moiety X)-type motif 14	EE806260.1	−3.048	−3.3985	−3.352
B4GALT2	UDP-Gal: betaGlcNAc beta 1,4- galactosyltransferase, polypeptide 2	DY491344.1	−3.364	1.924	1.649
ABCB9	ATP-binding cassette, sub-family B (MDR/TAP), member 9	EE818298.1	2.149	1.672	1.5765
KLK10	kallikrein related-peptidase 10	GO766063.1	−1.802	−1.3535	−1.302
SLC28A1	Na/nucleoside cotransporter	GO698122.1	−3.4035	−2.886	−3.101
-	-	EE834108.1	−3.603	−1.077	−1.697
C7H5ORF24	chromosome 5 open reading frame 24 ortholog	EE775125.1	1.289	1.301	1.2845
-	-	EE755431.1	−1.564	−1.9835	−2.1185
-	-	EE805398.1	2.4785	1.1985	1.0405
-	-	EE765998.1	−1.1025	1.6255	1.407
-	-	GO773148.1	−2.7225	2.127	2.1205
-	-	EE818179.1	−1.514	1.452	1.161
-	-	DY479153.1	−1.3705	1.4445	1.138
LOC100155914	similar to prion-like protein doppel	EE868622.1	−2.582	−1.266	−1.8615
-	-	DY491124.1	−2.879	−3.3315	−3.5585
-	-	XM_002696802.1	1.096	1.236	1.201
MCP1	mast cell proteinase-1	NM_001009472.1	1.05	2.9485	1.7575
SMAD4	SMAD family member 4	NM_001076209.1	−2.3525	−3.225	−3.2645
ZNF330	zinc finger protein 330	NM_001038157.1	−1.4705	2.286	1.784
SEPT7	septin 7	NM_001001168.1	−2.067	−1.758	−1.2665
COMMD4	COMM domain containing 4	NM_001040597.1	−2.3775	−1.203	−1.5445
-	-	XM_002696814.1	−1.526	−1.228	−1.203

### GO enrichment analysis

**Figure 1:**
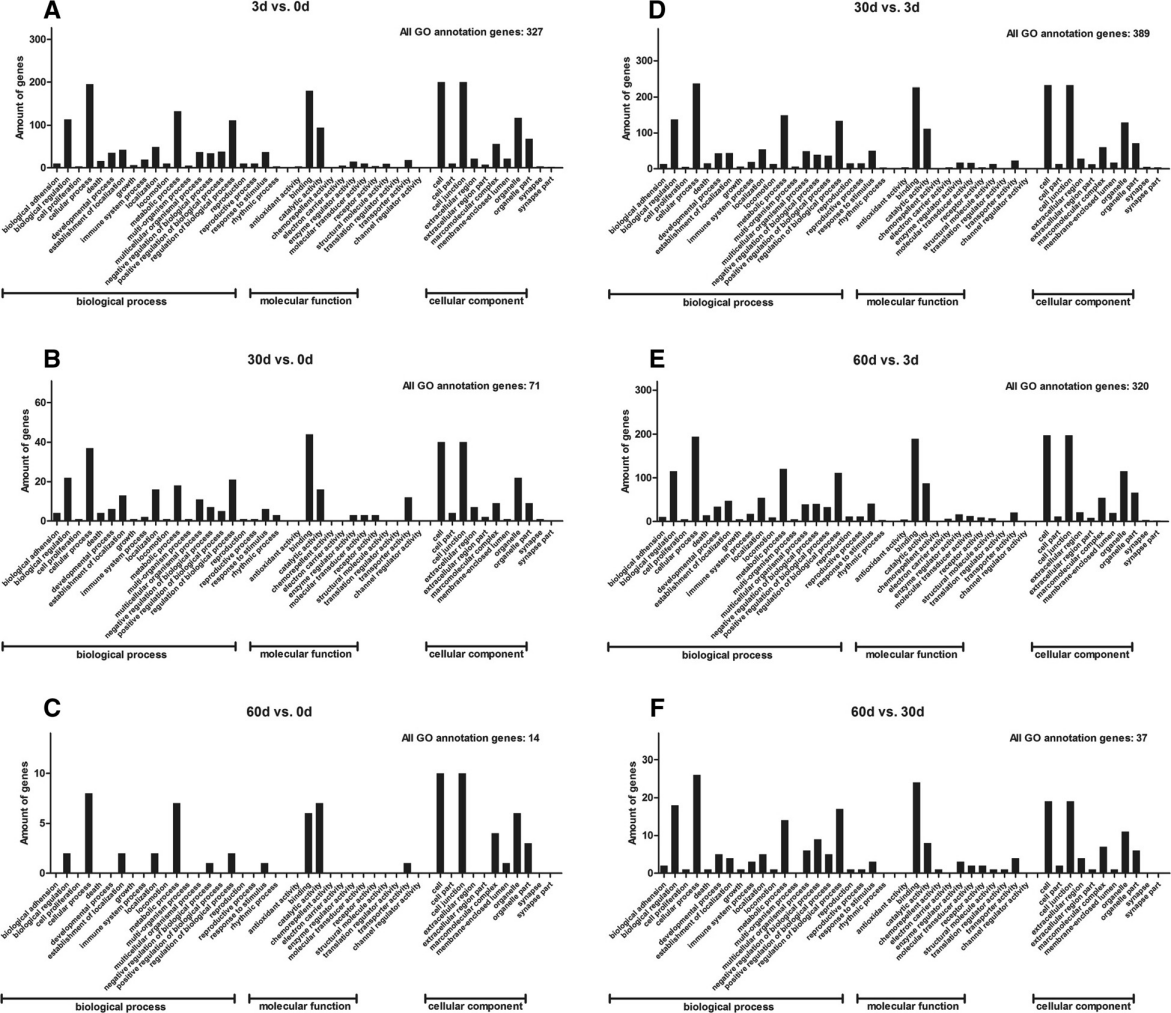
GO annotations of differentially expressed genes. Summary of functions and locations for genes differentially expressed in T lymphocytes of *H. contortus* infected sheep in six comparisons (**A**: 3d vs. 0d; **B**: 30d vs. 0d; **C**: 60d vs. 0d; **D**: 30d vs. 3d; **E**: 60d vs. 3d; **F**: 60d vs. 30d), grouped into the three GO subcategories ‘biological process’, ‘cellular component’ and ‘molecular function’.

In order to further elucidate the biological processes occurring in T lymphocytes immune responses in post *H. contortus* infection sheep, Gene Ontology (GO, www.geneontology.org/) Term Enrichment Analysis was performed to classify the differentially expressed genes into different functional categories. In this, 327, 71, 14, 389, 320 and 37 modulated genes acquired GO annotation in the six comparisons respectively (Table 2). Figure 1 shows the GO annotations of the six comparisons. Among them the main GO terms for differentially expressed genes were cellular processes, binding, cell, metabolic processes and biological regulation.

### KEGG pathway analysis

**Figure 2:**
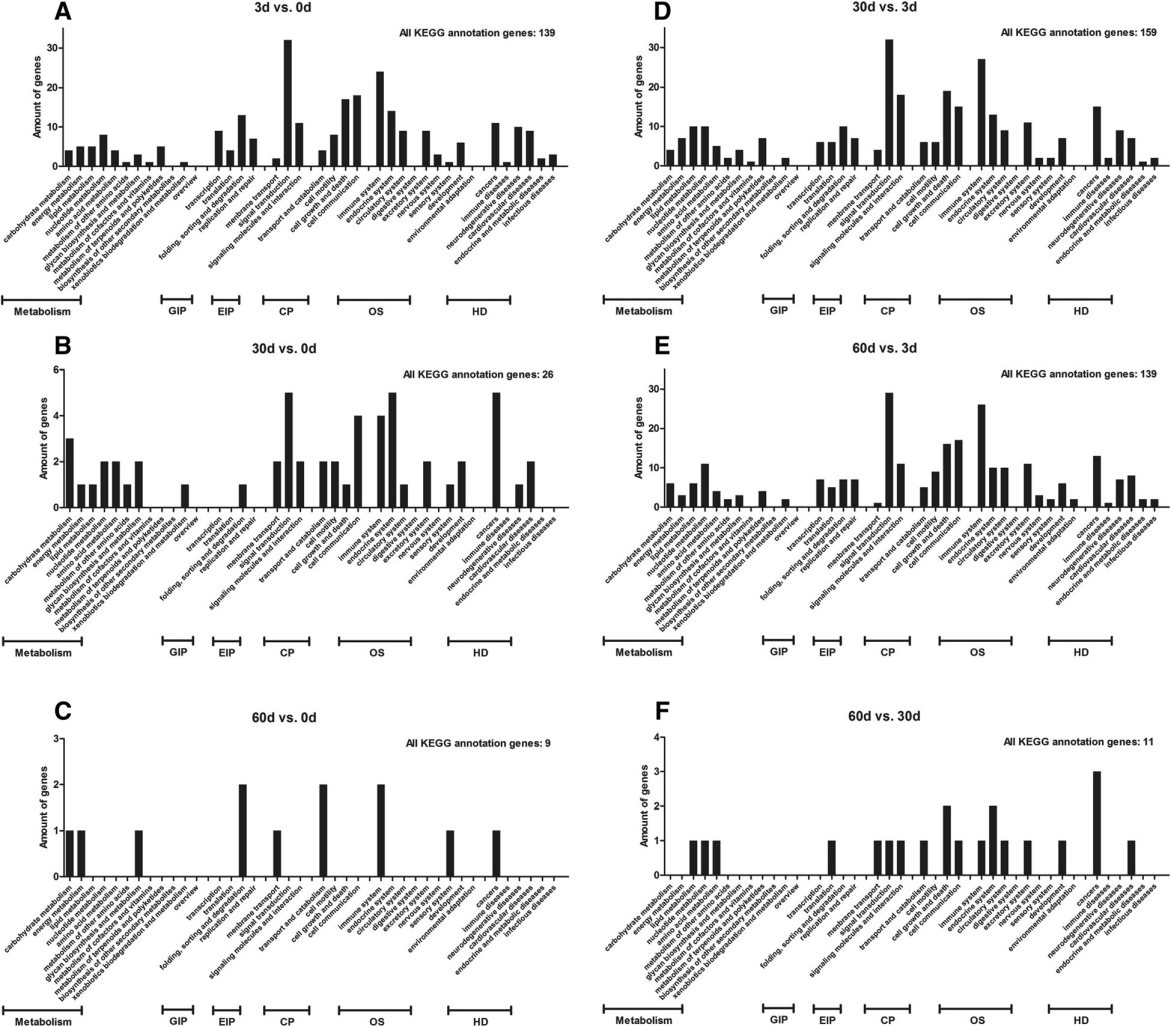
KEGG annotations of differentially expressed genes. All differentially expressed genes in six comparisons (**A**: 3d vs. 0d; **B**: 30d vs. 0d; **C**: 60d vs. 0d; **D**: 30d vs. 3d; **E**: 60d vs. 3d; **F**: 60d vs. 30d) were annotated using the KEGG database for pathway analysis, and were classified according to the six KEGG subcategories ‘metabolism’, ‘GIP (genetic information processing)’, ‘EIP (environmental information processing)’, ‘CP (cellular processes)’, ‘OS (organismal systems)’ and ‘HD (human diseases)’.

The KEGG pathway analysis (http://www.genome.jp/kegg/) was performed to determine the pathway analysis of all modulated genes. In this, 139, 26, 9, 159, 139 and 11 modulated genes acquired KEGG pathway annotations in the six comparisons respectively (Table 2). Figure 2 shows The KEGG pathway analysis in the six comparisons. The primary KEGG annotation terms were signal transduction, immune system, cell communication, cell growth and cell death.

Under ‘immune system’, the only immune-relevant KEGG pathway term, 24 (6 up-regulated), 4 (4 up-regulated), 2 (2 up-regulated), 27 (22 up-regulated), 26 (19 up-regulated) and 1 (0 up-regulated) modulated genes were found in the six comparisons respectively. These genes are listed in Table 4 and their details are listed in Additional file 2.

**Table 4:** **The correlative genes involved in the immune-relevant pathways from the results of KEGG pathway analysis in the six comparisons**

**Immune-relevant pathway**	**3d vs. 0d**	**30d vs. 0d**	**60d vs. 0d**	**30d vs. 3d**	**60d vs. 3d**	**60d vs.30d**
Hematopoietic cell lineage	CD59↓	-	*FCER2*↑	IL6R↑	IL1R2↑, CD59↑	-
Complement and coagulation cascades	CD59↓, C5AR1↓	-	-	C5AR1↑, C1R↑, CD59↑	C5AR1↑, CD59↑	-
Toll-like receptor signaling pathway	IRF7↓, MAP3K7IP2↓, MAPK9↓	IRAK1↑	-	IRAK1↑, MAP3K7IP2↑, MAPK9↑, IRF7↑	PIK3R2↑, MAPK9↑, IRF7↑	-
NOD-like receptor signaling pathway	*SUGT1*↓, MAP3K7IP2↓, MAPK9↓	-	-	MAP3K7IP2↑, MAPK9↑, *SUGT1*↑	MAPK9↑, *SUGT1*↑	-
RIG-I-like receptor signaling pathway	IRF7↓, LOC782671↓, MAPK9↓	-	-	LOC782671↑, MAPK9↑, IRF7↑	LOC782671↑, MAPK9↑, IRF7↑	-
Cytosolic DNA-sensing pathway	IRF7↓, LOC782671↓	-	-	LOC782671↑, IRF7↑	LOC782671↑, IRF7↑	-
Natural killer cell mediated cytotoxicity	*FCER1G*↓, PPP3R1↓	*SHC1*↑	-	PPP3R1↑, *SHC1*↑, *FCER1G*↑	PIK3R2↑, NFATC1↑, PPP3R1↑, *FCER1G*↑	*SHC1*↓
T cell receptor signaling pathway	PPP3R1↓, NCK2↑, *PAK4*↑	*PAK4*↑	-	NCK2↓, PPP3R1↑	*PAK4*↓, NCK2↓, PIK3R2↑, NFATC1↑, PPP3R1↑	-
B cell receptor signaling pathway	PPP3R1↓	-	-	PPP3R1↑	PIK3R2↑, NFATC1↑, PPP3R1↑	-
Fc epsilon RI signaling pathway	*FCER1G*↓, MAPK9↓	-	-	*FCER1G*↑, MAPK9↑	PIK3R2↑, MAPK9↑, *FCER1G*↑	-
Leukocyte transendothelial migration	CYBA↓, CYBB↑, MSN↓, CTNNB1↓, MYL9↑	-	-	CXCR4↓, MYL9↓, RHOH↓, CYBB↑	MYL9↓, RHOH↓, PIK3R2↑, CYBB↓, CYBA↑	-
Intestinal immune network for IgA production	TGFB1↓, PIGR↑, TGFB2↑	-	-	CXCR4↓, TGFB2↓, TGFB1↑	TGFB2↓, TGFB1↑	-
Cemokine signaling pathway	FOXO3↓, GNB2↓, *GSK3A*↓	*SHC1*↑, *GSK3A*↑	*GSK3A*↑	CXCR4↓, GNB2↑, FOXO3↑, *GSK3A*↑, *SHC1*↑	GNG10↓, PIK3R2↑, *GSK3A*↑, FOXO3↑, GNB2↑	*SHC1*↓
Fc gamma R-mediated phagocytosis	-	-	-	-	PIK3R2↑	-

An up or down arrow beside each gene indicates up- or down- regulated. Italic genes are also amongst top ten differentially expressed genes.

### Top up- or down-regulated genes

The top 10 up- or down-regulated genes in each of the six comparisons are listed in Additional file 3. Approximately half of these genes were uncharacterized, while the other half consisted of genes of known or noted hypothetical performances that include roles in immune response, protein metabolic process, sugar metabolic process and signaling transduction. Of particular interest are SUGT1, FCER1G, GSK3A, PAK4, SHC1 and FCER2, which are immune-related genes.

### Reverse transcription and quantitative RT-PCR (qPCR) validation

**Figure 3:**
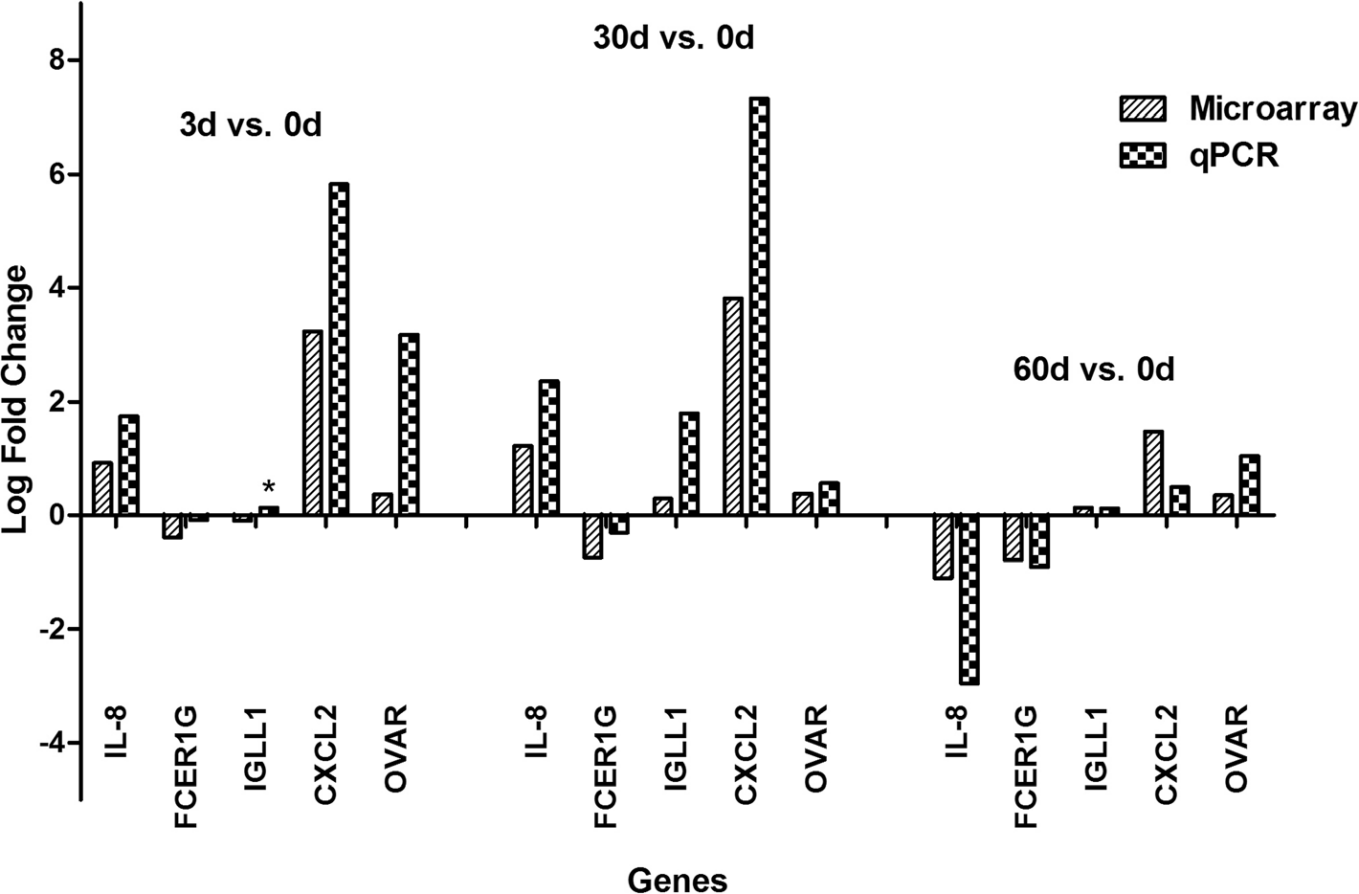
Validation of gene expression by quantitative RT-PCR. Five immune-relevant genes were randomly selected for qPCR validation. The values are the mean of gene expression levels performed three times, as calculated by the △Ct method, normalized to beta-actin expression, and comparing the 3d, 30d, 60d versus 0d of each sheep respectively. A similar degree of variability in expression profiles between microarray and qPCR was observed, only with IGLL1 opposite in 3d vs. 0d comparison. Opposite expression between qPCR and microarray was marked by an asterisk.

To verify the microarray data, qPCR was performed on five randomly selected immune-relevant genes which either showed differential expression in microarray analysis or related to immune response (IL-8, FCER1G, IGLL1, CXCL2 and OVAR). Fold changes were normalized to beta-actin expression that served as an internal control. As shown in Figure 3, the qPCR results are consistent with the microarray data. However it is important to note that albeit displaying similar trends, the fold changes in qPCR results does not quite match to that as revealed by the microarray experiment.

## Discussion

In this experiment, we infected sheep with a single bolus dose of *H. contortus* larvae to simulate a primary infection. While a trickle infection challenge would be a better strategy to mirror field-acquired immunity, mimicking a mature individual developing immune response at a natural speed, our approach and data, on the other hand reflect a much more direct response to infection in sheep, including initial innate immune responses, followed by adaptive immune response to surviving worms.

As the sheep genome has yet to be sequenced, we used microarray composed of ovine sequences and useful sequences from other species, to identify genes that were significantly differentially expressed in the various stages in sheep T lymphocytes upon *H. contortus* infection. Our results show that the number of modulated genes decreased with post infection day, inferring that *H. contortus* had eventually achieved homeostasis with the host via immune evasion. This inference is supported by the fecal egg count (FEC) during *H. contortus* infection and the final abomasal necropsy, where FEC peaked at 2,400 eggs per gram (data not shown) while only a few adult nematodes were found at the point of abomasal necropsy, even though each sheep was infected with as many as 17,000 L3s. This drop was a significant reduction.

The bulk of modulated genes in the 3d vs. 0d, 60d vs. 0d, and 60d vs. 30d comparisons were down-regulated genes. However majority of those in the 30d vs. 0d, 30d vs. 3d and 60d vs. 3d comparisons were up-regulated (Table 2). When sheep are being challenged by a large number of L3s, the larvae migrate, grow up to blood-feeding L4s and begin to invade the host system within a very short time. During this early stage of infection, larvae infiltrated the abomasal crypts and released a substantial arsenal of secreted products [27]. These products with known roles in tissues degradation and possible immune-modulatory roles are likely to circulate through systematic circulation, destroying immune cells including T lymphocytes, in an effort to assist larvae development and evasion [28]. In addition, the dose of 17,000 worms is considered to be an enormous amount for nematode-naive sheep and represents a dose that can lead to host fatality. As such, it is possible that the initial transcript patterns reflect the physical and physiological trauma that the animal was experiencing. Thus, down-regulated genes accounted for most of the modulated genes in the 3d vs. 0d comparison. At 25–30 dpi, the *H. contortus* larvae that failed to be expelled from the host system would now present as adults. This status depends delicately on the interactions between the parasites and their host [27]. Female adults were fully gravid by now after copulation with the males. While the adults continued to grow, the sheep began to mount a stronger T lymphocyte immune response against these parasites and the millions of egg produced [7]. Thus, differentially expressed genes related to immune system were mostly up-regulated genes in the 30d vs. 0d and 30d vs. 3d comparisons. However, more down, than up-regulated genes were observed at 60 dpi. This observation could be due to a strong wave of immune response elicited in the middle of the infection that has brought about a decreased worm burden during the later stage of infection. Only about one hundred adults were (data not shown) found in the abomasums when the sheep were autopsied at 60 dpi. These remaining parasites managed to survive from the host immune responses by immune-escape and eventually achieving homeostasis within its host.

To investigate the modulated genes in greater detail, we applied GO annotation for gene clustering. About half of the genes are clustered into one or more than one GO terms. The most frequent GO terms are binding, cellular process, biological regulation and metabolic process. To function optimally, T lymphocytes perform certain housekeeping tasks such as protecting cell structure from parasite attack and maintaining intracellular biological regulation [29]. In association with these protective measures against parasite invasion, many genes involved in cellular homeostasis maintenance were differentially expressed throughout the infection (Additional file 1). Several genes help in the communication between T lymphocytes and other immune cells and/or antigens while others assist in cell metabolism. This clustering data revealed a strong relationship between sheep immune response and cellular homeostasis.

Interestingly, there are only fourteen known genes among the thirty-five genes shared in the 3d vs. 0d, 30d vs. 0d and 60d vs. 0d comparisons, including SLC9A3R2, SCN8A, B4GALT2, ZNF330, ABCB9, MCP1, RXRB, C7H5ORF24, COMMD4, NUDT14, KLK10, SLC28A1, SMAD4, and SEPT7. SLC9A3R2 encodes the sodium-hydrogen exchange regulator confactor NHE-RF2, which generally interacts with a sodium/hydrogen exchanger NHE3 that is primarily responsible for transepithelial sodium balance to maintain endothelial homeostasis [30]. Decreased transcription of SLC9A3R2 at 3 dpi may reflect an initial physical trauma experienced from an enormous infection dose, which gave rise to an imbalance intracellular sodium level. A subsequent increased transcriptional activity at 30 dpi and 60 dpi helped to recover the disturbed salt balance and provided signals to stimulate chemokine generation and NF-κB activation for the regulation of a series of immunological responses [31]. SCN8A encodes for a protein that modulates sodium balance as well. It is the alpha subunit of a voltage-gated sodium channel, forming ion conduction pore to produce a functional channel. The beta and alpha subunits work together to regulate channel gating [32,33]. Like SLC9A3R2, SCN8A down and up-regulation at 3 dpi and the two later time points respectively also indicated sodium balance regulation during infection. ZNF330, a highly conserved protein, was first characterized immunologically as a human autoantigen [34]. It has been demonstrated that this zinc finger protein is capable of mediating mitochondrial apoptotic pathway and directing specific proteins to the nucleolus to regulate nucleolar transcription [35]. ZNF330 displayed a similar expression profile and function as B4GALT2. B4GALT2 is a member of the beta-1,4-galactosyltransferase gene family. As membrane-bound glycoproteins, members in the family direct proteins to the Golgi apparatus and remain as a transmembrane anchor [36]. It is undeniable that *H. contortus* infection induces cellular damage in sheep. Reduced transcription of B4GALT2 and ZNF330 at 3 dpi was consistent with the inability of protein transportation between or within T lymphocytes, later where cells tried to activate apoptosis to achieve homeostasis by increasing B4GALT2 and ZNF330 levels at 30 and 60 dpi. [37]. ABCB9 is a member of the MDR/TAP subfamily of ATP-binding cassette (ABC) transporters. Though the function of the transporter has yet to be determined, ABCB1, another member of the MDR/TAP subfamily, shows correlation with migration of immune cells and adaptive immunity [38,39]. Fujimoto speculated that more ABCB9 might help to regulate antigen presentation and multidrug resistance [40]. Increased expression of MCP1 encoded mast cell protease 1 would contribute to the clearance of gastrointestinal nematode [41]. Mast cell proteases are protective mediators of inflammation [42]. Up-regulated levels of ABCB9 and MCP1 during infection help the host to mobilize immune cells and present antigen against *H. contortus* infection. A higher transcription level of RXRB encoding retinoid X receptor beta, a nuclear receptor, modulates cell growth and differentiation through increasing transcriptional function and DNA binding [43]. The gene maps to the MHC class II regions [44]. C7H5ORF24, a bovine ortholog of human chromosome 5 open reading frame, acts as a cancer marker to address DNA damage response [45,46]. The up-regulation of RXRB and C7H5ORF24 at the three time points indicated modulation of cell development and repair during T lymphocytes homeostasis maintenance. COMMD4, down-regulated in the three comparisons, is a protein widely conserved throughout evolution and involved in NF-κB termination [47-49]. NF-κB plays a key role in cellular responses to stimuli, as a rapid-acting primary transcription factor. Κ light chains of NF-κB are also key components of immunoglobulins, making NF-κB an important humoral immune modulator. The factor was activated to up-regulate genes associated with T-cell development and humoral immune response when the level of COMMD4 is low. Misregulation of NF-κB could possibly lead to cancer, viral infection and inadequate immune development. In brief, the protein complex finally manifests as a cytokine production and cell survival factor [50]. Decreased expression of NUDT14 encoding for UDP-glucose pyrophosphatases (UGPPase) would influence the synthesis of glycogen from glucose (UDP-glucose metabolism) [51]. Methylation of KLK10 would be a novel prognostic marker of cancers [52]. A lower level of KLK10 limited the activity of tumorigenesis. CNT1, expressed by SLC28A1, is down-regulated that would affect nucleotide biosynthesis in nucleoside salvage pathways and pyrimidine-nucleoside transportation [53]. SMAD4, down-regulated during infection, binds receptor-regulated SMADs to serve as a transcription activator that regulates TGF-beta receptor-mediated signaling [54]. SEPT7 encoded protein is highly similar to yeast CDC10, which is localized to the cytoplasmic membrane and is an absolute requirement for the completion of cytokinesis. However, decreased SEPT7 expression has no effect on mitosis in T lymphocytes, which suggest that this septin may not be the key molecule even though it is able to destabilize microtubule [55]. Lower transcription levels of NUDT14, KLK10, SLC28A1, SMAD4 and SEPT7 during a series of stages of infection may reflect the inabilities of these genes to participate in glucose metabolism, tumorigenesis, nucleoside biosynthesis and transport, signaling and microtubule stabilization respectively. However other supplementary genes, such as C7H5ORF24 mentioned above, were able to perform similar functions to maintain T lymphocyte homeostasis. Down-regulation of SLC9A3R2, SCN8A, B4GALT2, ZNF330, NUDT14, KLK10, SLC28A1, SMAD4 and SEPT7 in the 3d vs. 0d comparison suggested a strong homeostasis regulation during the early stage of infection. This is likely to be contributed by the initial trauma induced by an enormous infection dose used in our experiments. Twenty-one genes remained unknown. Our data implies that maintaining both an effective immunological response and a constant cellular environment within the host is of equal importance during T lymphocytes battle against *H. contortus* infection.

Differentially expressed genes were also subjected to KEGG pathway analysis to obtain a more systematic functional annotation. In addition to signaling, cell communication and cell growth and death, KEGG annotations locate more genes to immune system. These genes contribute to almost all immunological processes. Of particular interest are SUGT1, PAK4, FCER1G, GSK3A, SHC1 and FCER2. These genes are also amongst the top ten differentially expressed genes. PAK4 serves as an enzyme in the family of serine/threonine p21-activating kinases. A higher expression of PAK4 at 3 dpi and 30dpi may suggest T cell activation and inflammation mediated by chemokine and cytokine signaling pathway [56,57]. PAK proteins play a role in cytoskeleton reorganization and nuclear signaling and have been implicated in a large-scale of biological activities. PAK4 is also a critical effector that link Rho GTPases to actin cytoskeleton reorganization. Furthermore, this kinase may act as a factor to support cytoskeleton organization of T lymphocytes. The up-regulation of SUGT1 enhanced NOD-like receptor activation [58]. The NOD-like receptors are involved in inflammation regulation through the activation of IL-1 and the NF-κB signaling pathway, meanwhile the proteins also regulate apoptotic responses. Depletion of SUGT1 prevents multiple cellular responses associated with Nod1 activation, but did not affect Nod1 protein stability. Heat shock proteins are binding partners of the SUGT1 protein, involved in innate immune responses [59]. Stronger inflammatory responses are suggested in T lymphocytes at 30 dpi by greater SUGT1 and PAK4 expression compared to that at 0 dpi. GSK3A is a multi-task serine kinase with a role in phosphoinositide 3-kinase (especially PIK3CG, which regulates cytotoxicity in NK cells) signaling pathway and chemokine signaling pathway [60,61]. The up-regulated glycogen synthase kinase modulates various transcription factors, as well as embryo development. However the kinase seems to be non-essential to vitality, as GSK3A mutant mice showed no significant abnormalities. There are three SHC1 protein isoforms. These isoforms differ in activity and domain structures, showing vital functions in various aspects. A higher level of SHC1 delivers a strong signal for chemokine and natural killer cell mediated cytotoxicity signaling pathway [62-64]. When SHC1 acts as a dynamic scaffold protein in cell surface receptor, they may regulate cell invasion and cytoskeleton reorganization. The proteins also operate in the regulation of apoptosis and tumorigenicity in mammalian cells. GSK3A and SHC1 levels were increased at 30 dpi and 60 dpi. They co-regulate chemokine signaling pathways while mediate other immune-relevant signaling pathways separately. The four genes mentioned above exhibit roles in both immunological and cellular processes in T lymphocytes. FCER1G is the gamma chain of high-affinity receptor for the Fc region of IgE. Up-regulated FCER1G directs mast cell and coupling allergens to suppress inflammatory and immediate hypersensitivity responses in immunity to parasites and in allergic diseases [65-67]. Another receptor for IgE, FCER2 (CD23), also known as the low-affinity receptor, is involved in resistance to parasites and is also found to be up-regulated in this study. CD23 molecules regulate IgE levels, and then bind to the IgE immune complexes on B cells to activate macrophages and eosinophils [68]. Upon *H. contortus* infection, secretions from nematodes enter the blood stream of the sheep and are captured by IgE. The antigen is then transferred from CD23+ B cells to CD4+ T cells via CD11c + antigen presenting cells, leading to an intensive antibody response [69]. FCER1G cooperates with FCER2 to enhance host humoral immune responses, which are reflected by their up-regulations at 30 dpi and 60 dpi.

Analysis of the top ten differentially expressed genes revealed a big increase in the expression of IL-13 in the 60d vs. 30d comparison. Most cytokine kinetics studies have reported early up-regulation in IL-13 mRNA levels by 3dpi, peak at 7dpi followed by a gradual decline until 30dpi. IL-13, one of the key cytokines in Th2-type immune response, induces IgE secretion that in turn, guiding immune cells to kill nematodes [70]. Yet a striking discovery in our experiment shows a recovery in IL-13 mRNA levels by 60 dpi. We speculate that this interesting observation may imply a final “mop-up” phenomenon to eliminate all nematodes at the final stage of sheep immunity against *H. contortus* infection.

### Ethical approval

The data reported in our manuscript was collected from animals. The ethics was approved by the experimental animal ethics committee of Zhejiang University and the reference number is ZJU201308-1-10-072.

## Conclusions

In summary, the present study sought to describe changes in gene expression across various time points during the infection period of sheep T lymphocytes by *H. contortus* using microarrays and modern bioinformatics analysis. A list of differentially expressed genes having roles in homeostasis maintenance and immune response was obtained, together with many other genes of unknown function. Changes occurring in the sheep T lymphocytes at the molecular level correspond well to the general description of nematode immunity at the cellular level. The elaborate list of candidate genes obtained here provides new research directions for the study of long-term resistance to *H. contortus* infection in sheep. Future work includes the identification of novel *H. contortus* infection resistance genes in sheep.

## Additional files

Additional file 1:
**Details on differentially expressed genes in the six comparisons.** Detail information of all modulated genes was listed according to probe ID, log2 fold change, p value, gene description, gene symbol and so on. Additional file 2:
**Immune-relevant genes from KEGG pathway analysis of differentially expressed genes in the six comparisons.** Symbol, name, GenBank No. and log fold change of immune-relevant genes were listed. Additional file 3:
**Top ten up-regulated and down-regulated genes in the six comparisons.** Symbol, name, GenBank No. and log fold change of top ten genes were listed. About half genes were yet to be identified.
