# Supplementary material for: Profiling of differentially expressed genes in sheep T lymphocytes response to an artificial primary Haemonchus contortus infection
Source: Parasit Vectors. 2015 Apr 18;8:235. doi: 10.1186/s13071-015-0844-z (PMC4406218; doi:10.1186/s13071-015-0844-z)
Supplement: Additional file 2: — Immune-relevant genes from KEGG pathway analysis of differentially expressed genes in the six comparisons. Symbol, name, GenBank No. and log fold change of immune-relevant genes were listed. [file 13071_2015_844_MOESM2_ESM.pdf]

Additional file 2 Immune-relevant genes from KEGG pathway analysis of differentially expressed genes in six comparisons.

Gene symbol	Gene name	GenBank #	Log fold change
3d vs. 0d			
FCER1G	Fc fragment of IgE, high affinity I, receptor for; gamma polypeptide	NM_174537.2	-6.827
SUGT1	SGT1, suppressor of G2 allele of SKP1 (*S. cerevisiae*)	NM_001046203.1	-5.5395
IRF7	interferon regulatory factor 7	NM_001105040.1	-3.9895
CYBA	cytochrome b-245, alpha polypeptide	NM_174034.2	-3.543
CD59	CD59 molecule, complement regulatory protein	Oar#S52818900	-2.3505
PPP3R1	protein phosphatase 3 (formerly 2B), regulatory subunit B, alpha isoform	NM_000945.3	-2.129
FOXO3	forkhead box O3; forkhead box O3B pseudogene	NM_001455.3	-2.1155
CCYBB	cytochrome b-245, beta polypeptide	NM_007807.4	-1.712
MAP3K7IP2	mitogen-activated protein kinase kinase kinase 7 interacting protein 2	Oar#S35009065	-1.5625
MAVS	similar to Virus-induced signaling adapter; mitochondrial antiviral signaling protein	NM_001046620.1	-1.526
MAPK9	mitogen-activated protein kinase 9	NM_001046369.1	-1.446
C5AR1	complement component 5a receptor 1	NM_001007810.3	-1.4185
GNB2	guanine nucleotide binding protein (G protein), beta polypeptide 2	NM_001097561.1	-1.3725
GSK3A	glycogen synthase kinase 3 alpha	NM_001102192.1	-1.3545
TGFB1	transforming growth factor, beta 1	NM_214015.1	-1.1845
MSN	moesin	NM_001046477.1	-1.12
MAVS	similar to Virus-induced signaling adapter; mitochondrial antiviral signaling protein	NM_001046620.1	-1.0405
CTNNB1	catenin (cadherin-associated protein), beta 1, 88kDa	NM_001076141.1	-1.0405
CYBB	cytochrome b-245, beta polypeptide	NM_174035.3	1.044
NCK2	NCK adaptor protein 2	NM_001099036.1	1.076
PIGR	polymeric immunoglobulin receptor	NM_174143.1	1.2755
TGFB2	transforming growth factor, beta 2	NM_001113252.1	1.3695
MYL9	myosin, light chain 9, regulatory	NM_001075234.1	1.375

PAK4	p21 protein (Cdc42/Rac)-activated kinase 4	NM_001076184.1	2.135
30d vs. 0d			
IRAK1	interleukin-1 receptor-associated kinase 1	NM_001040555.1	1.073
PAK4	p21 protein (Cdc42/Rac)-activated kinase 4	NM_001076184.1	1.406
SHC1	SHC (Src homology 2 domain containing) transforming protein 1	NM_001164061.1	2.638
GSK3A	glycogen synthase kinase 3 alpha	NM_019884.2	2.7745
60d vs. 0d			
CD23	Fc fragment of IgE, low affinity II, receptor for (CD23)	NM_001081807.1	1.3945
GSK3A	glycogen synthase kinase 3 alpha	NM_019884.2	2.105
30d vs. 3d			
CXCR4	chemokine (C-X-C motif) receptor 4	NM_174301.3	-1.4115
MYL9	myosin, light chain 9, regulatory	NM_001075234.1	-1.3825
TGFB2	transforming growth factor, beta 2	NM_001113252.1	-1.266
RHOH	ras homolog gene family, member H	NM_004310.3	-1.1015
NCK2	NCK adaptor protein 2	NM_001099036.1	-1.059
IL6R	interleukin 6 receptor	NM_000565.2	1.113
MAVS	similar to Virus-induced signaling adapter; mitochondrial antiviral signaling protein	NM_001046620.1	1.136
IRAK1	interleukin-1 receptor-associated kinase 1	NM_001040555.1	1.1915
IRAK1	interleukin-1 receptor-associated kinase 1	NM_001040555.1	1.2465
MAP3K7IP2	mitogen-activated protein kinase kinase kinase 7 interacting protein 2	Oar#S35009065	1.273
MAPK9	mitogen-activated protein kinase 9	NM_001046369.1	1.3
C5AR1	complement component 5a receptor 1	NM_001007810.3	1.3425
CYBB	cytochrome b-245, beta polypeptide	NM_007807.4	1.394
GNB2	guanine nucleotide binding protein (G protein), beta polypeptide 2	NM_001097561.1	1.5855
C1R	complement component 1, r subcomponent	NM_001034407.1	1.604
FOXO3	forkhead box O3; forkhead box O3B pseudogene	NM_001455.3	1.812

TGFB1	transforming growth factor, beta 1	NM_214015.1	1.8785
MAVS	similar to Virus-induced signaling adapter; mitochondrial antiviral signaling protein	NM_001046620.1	2.1635
PPP3R1	protein phosphatase 3 (formerly 2B), regulatory subunit B, alpha isoform	NM_000945.3	2.193
CD59	CD59 molecule, complement regulatory protein	Oar#S52818900	2.332
GSK3A	glycogen synthase kinase 3 alpha	NM_001102192.1	2.3915
SHC1	SHC (Src homology 2 domain containing) transforming protein 1	NM_001164061.1	2.5275
GSK3A	glycogen synthase kinase 3 alpha	NM_019884.2	2.9
CYBA	cytochrome b-245, alpha polypeptide	NM_174034.2	3.7895
IRF7	interferon regulatory factor 7	NM_001105040.1	4.0675
SUGT1	SGT1, suppressor of G2 allele of SKP1 (*S. cerevisiae*)	NM_001046203.1	5.236
FCER1G	Fc fragment of IgE, high affinity I, receptor for; gamma polypeptide	NM_174537.2	6.9725
60d vs. 3d			
TGFB2	transforming growth factor, beta 2	NM_001113252.1	-1.4945
MYL9	myosin, light chain 9, regulatory	NM_001075234.1	-1.4225
PAK4	p21 protein (Cdc42/Rac)-activated kinase 4	NM_001076184.1	-1.2695
RHOH	ras homolog gene family, member H	NM_004310.3	-1.122
GNG10	guanine nucleotide binding protein (G protein), gamma 10	NM_001114512.1	-1.0695
CYBB	cytochrome b-245, beta polypeptide	NM_174035.3	-1.057
NCK2	NCK adaptor protein 2	NM_001099036.1	-1.0195
PIK3R2	phosphoinositide-3-kinase, regulatory subunit 2 (beta)	NM_174576.2	1.1
NFATC1	nuclear factor of activated T-cells, cytoplasmic, calcineurin-dependent 1	NM_172388.1	1.107
MAVS	similar to Virus-induced signaling adapter; mitochondrial antiviral signaling protein	NM_001046620.1	1.156
TGFB1	transforming growth factor, beta 1	NM_214015.1	1.164
IL1R2	interleukin 1 receptor, type II	NM_001046210.1	1.1925
C5AR1	complement component 5a receptor 1	NM_001007810.3	1.415
PPP3R1	protein phosphatase 3 (formerly 2B), regulatory subunit B, alpha isoform	NM_000945.3	1.4185

CYBB	cytochrome b-245, beta polypeptide	NM_007807.4	1.442
MAPK9	mitogen-activated protein kinase 9	NM_001046369.1	1.4735
MAVS	similar to Virus-induced signaling adapter; mitochondrial antiviral signaling protein	NM_001046620.1	1.826
GSK3A	glycogen synthase kinase 3 alpha	NM_001102192.1	1.883
FOXO3	forkhead box O3; forkhead box O3B pseudogene	NM_001455.3	1.897
GNB2	guanine nucleotide binding protein (G protein), beta polypeptide 2	NM_001097561.1	1.897
CD59	CD59 molecule, complement regulatory protein	Oar#S52818900	2.164
GSK3A	glycogen synthase kinase 3 alpha	NM_019884.2	2.2305
IRF7	interferon regulatory factor 7	NM_001105040.1	3.7835
CYBA	cytochrome b-245, alpha polypeptide	NM_174034.2	3.8565
SUGT1	SGT1, suppressor of G2 allele of SKP1 (*S. cerevisiae*)	NM_001046203.1	5.201
FCER1G	Fc fragment of IgE, high affinity I, receptor for; gamma polypeptide	NM_174537.2	7.172
60d vs. 30d			
SHC1	SHC (Src homology 2 domain containing) transforming protein 1	NM_001164061.1	-2.892
