# Supplementary material for: Profiling of differentially expressed genes in sheep T lymphocytes response to an artificial primary Haemonchus contortus infection
Source: Parasit Vectors. 2015 Apr 18;8:235. doi: 10.1186/s13071-015-0844-z (PMC4406218; doi:10.1186/s13071-015-0844-z)
Supplement: Additional file 3: — Top ten up-regulated and down-regulated genes in the six comparisons. Symbol, name, GenBank No. and log fold change of top ten genes were listed. About half genes were yet to be identified. [file 13071_2015_844_MOESM3_ESM.pdf]

Additional file 3 Top ten up-regulated and down-regulated genes in the six comparisons.

Gene symbol	Gene name	GenBank #	Log fold change
3d vs. 0d			
FCER1G	Fc fragment of IgE, high affinity I, receptor for; gamma polypeptide	NM_174537.2	-6.827
DNAJB4	DnaJ (Hsp40) homolog, subfamily B, member 4	EE780217.1	-6.501
-	-	EE807798.1	-6.259
-	-	DY506357.1	-5.7985
SUGT1	SGT1, suppressor of G2 allele of SKP1 (*S. cerevisiae*)	NM_001046203.1	-5.5395
NAB2	NGFI-A binding protein 2 (EGR1 binding protein 2)	NM_001045897.1	-5.533
ASZ1	ankyrin repeat, SAM and basic leucine zipper domain containing 1	NM_001114141.1	-5.376
-	-	CU638271.1	-5.16
-	-	EE872292.1	-4.8505
-	-	FE036121.1	-4.79
-	-	EE795074.1	1.683
PPP2CB	protein phosphatase 2 (formerly 2A), catalytic subunit, beta isoform	NM_001009552.1	1.879
-	-	Oar#S34883954	1.9005
ATP1A1,	ATPase, Na+/K+ transporting, alpha 1 polypeptide	NM_001009360.1	1.9335
PAK4,	p21 protein (Cdc42/Rac)-activated kinase 4	NM_001076184.1	2.135
ABCB9	ATP-binding cassette, sub-family B (MDR/TAP), member 9	NM_022238.1	2.149
SAP30L	SAP30-like	EE801910.1	2.275
-	-	Oar#S20545387	2.3505
-	-	EE805398.1	2.4785
-	-	EE795477.1	3.1315
30d vs. 0d			
-	-	GO684492.1	-1.444
-	-	GO679819.1	-1.332

LOC100155914	similar to prion-like protein doppel	EE868622.1	-1.266
-	-	EE867875.1	-1.206
COMMD4	COMM domain containing 4	NM_001040597.1	-1.203
GUF1	GUF1 GTPase homolog (*S. cerevisiae*); similar to GUF1 GTPase homolog	NM_001102067.1	-1.1365
-	-	EE834108.1	-1.077
-	-	GO758506.1	-1.005
CIDEA	cell death-inducing DFFA-like effector a	NM_001083449.1	2.865
SLC9A3R2	solute carrier family 9 (sodium/hydrogen exchanger), member 3 regulator 2	NM_001137639.1	2.878
-	-	Oar#S34897158	2.919
-	-	EE869968.1	3.482
TMEM25	transmembrane protein 25	NM_001083639.1	4.133
2900056M07Rik	RIKEN cDNA 4922502B01 gene	EE812503.1	4.2675
BCAR3	breast cancer anti-estrogen resistance 3	NM_001024483.1	4.3505
-	-	Oar#S20553143	4.5415
-	-	DY495117.1	4.7625
-	-	GO684733.1	6.433
60d vs.0d			
LOC100155914	similar to prion-like protein doppel	EE868622.1	-1.8615
-	-	EE868723.1	-1.7305
-	-	EE834108.1	-1.697
ASB8	ankyrin repeat and SOCS box-containing 8	NM_001076452.1	-1.6595
-	-	DY506357.1	-1.6045
-	-	Oar#S34960908	-1.5735
PSMA3	proteasome (prosome, macropain) subunit, alpha type, 3	NM_001034235.1	-1.545
COMMD4	COMM domain containing 4	NM_001040597.1	-1.5445
NACC1	nucleus accumbens associated 1, BEN and BTB (POZ) domain containing	GO682032.1	-1.529

A6QPN8	hypothetical LOC522763; similar to Myeloid-associated differentiation marker	NM_001101956.1	-1.485
LOC443162	galectin-14	NM_001009251.1	1.3005
CD23	Fc fragment of IgE, low affinity II, receptor for (CD23)	NM_001081807.1	1.3945
ABCB9	ATP-binding cassette, sub-family B (MDR/TAP), member 9	NM_022238.1	1.5765
B4GALT2	UDP-Gal:betaGlcNAc beta 1,4- galactosyltransferase, polypeptide 2	NM_001105030.1	1.649
-	-	EE823315.1	2.0075
GSK3A	glycogen synthase kinase 3 alpha	NM_019884.2	2.105
-	-	GO773148.1	2.1205
-	-	GO782131.1	2.1525
SLC9A3R2	solute carrier family 9 (sodium/hydrogen exchanger), member 3 regulator 2	NM_001137639.1	2.3875
-	-	DY495117.1	3.9485
30d vs. 3d			
-	-	EE782785.1	-2.1515
-	-	EE795477.1	-1.994
ATP1A1	ATPase, Na+/K+ transporting, alpha 1 polypeptide	NM_001009360.1	-1.838
SAP30L	SAP30-like	EE801910.1	-1.7955
-	-	Oar#S20545387	-1.7495
-	-	EE795074.1	-1.729
LOC100155171	similar to Poly [ADP-ribose] polymerase 15 (PARP-15) (B-aggressive lymphoma protein 3)	GO709895.1	-1.6915
-	-	Oar#S34967559	-1.618
ZNF7	similar to zinc finger protein 7 (KOX 4, clone HF.16)	GO736953.1	-1.598
-	-	Oar#S34883954	-1.568
-	-	GO773148.1	4.8495
TMEM25	transmembrane protein 25	NM_001083639.1	4.919
-	-	CU638271.1	4.924
NAB2	NGFI-A binding protein 2 (EGR1 binding protein 2)	NM_001045897.1	5.083

SUGT1	SGT1, suppressor of G2 allele of SKP1 (*S. cerevisiae*)	NM_001046203.1	5.236
B4GALT2	UDP-Gal:betaGlcNAc beta 1,4- galactosyltransferase, polypeptide 2	NM_001105030.1	5.288
-	-	EE807798.1	5.9205
DNAJB4	DnaJ (Hsp40) homolog, subfamily B, member 4	EE780217.1	6.329
FCER1G	Fc fragment of IgE, high affinity I, receptor for; gamma polypeptide	NM_174537.2	6.9725
-	-	GO684733.1	7.7695
60d vs. 3d			
-	-	EE795074.1	-2.52
-	-	EE795477.1	-2.4945
-	-	Oar#S20545387	-2.3225
HBB	hemoglobin, gamma; hemoglobin, gamma 2; hemoglobin, beta	NM_173917.2	-2.1285
-	-	Oar#S34967559	-1.9595
SAP30L	SAP30-like	EE801910.1	-1.873
MYLK	myosin light chain kinase	NM_176636.2	-1.76
LOC615685	UPF0452 protein C7orf41 homolog	NM_001101246.1	-1.693
MMRN2	elastin microfibril interfacer 3	Oar#S20543407	-1.6445
LOC100155171	similar to Poly [ADP-ribose] polymerase 15 (PARP-15) (B-aggressive lymphoma protein 3)	GO709895.1	-1.6055
-	-	EE872292.1	4.64
ASZ1	ankyrin repeat, SAM and basic leucine zipper domain containing 1	NM_001114141.1	4.811
-	-	CU638271.1	4.811
-	-	GO773148.1	4.843
NAB2	NGFI-A binding protein 2 (EGR1 binding protein 2)	NM_001045897.1	4.927
B4GALT2	UDP-Gal:betaGlcNAc beta 1,4- galactosyltransferase, polypeptide 2	NM_001105030.1	5.013
DNAJB4	DnaJ (Hsp40) homolog, subfamily B, member 4	EE780217.1	5.1845
SUGT1	SGT1, suppressor of G2 allele of SKP1 (*S. cerevisiae*)	NM_001046203.1	5.201
-	-	EE807798.1	5.4765

FCER1G	Fc fragment of IgE, high affinity I, receptor for; gamma polypeptide	NM_174537.2	7.172
60d vs. 30d			
-	-	GO684733.1	-6.2025
-	-	Oar#S20553143	-5.3875
TMEM25	transmembrane protein 25	NM_001083639.1	-5.347
2900056M07Rik	RIKEN cDNA 4922502B01 gene	EE812503.1	-4.666
BCAR3	breast cancer anti-estrogen resistance 3	NM_001024483.1	-4.6565
-	-	EE869968.1	-4.057
-	-	Oar#S34897158	-3.162
-	-	Oar#S34886470	-2.996
-	-	Oar#S20553586	-2.963
SHC1	SHC (Src homology 2 domain containing) transforming protein 1	NM_001164061.1	-2.892
CDK6	cyclin-dependent kinase 6	NM_001259.6	1.0415
IL13	interleukin 13	NM_001082594.1	1.2155
